# Key Elements of Effective and Practical Disclosure Policies for Health Science Journals

**DOI:** 10.1289/ehp.12620

**Published:** 2009-06

**Authors:** Jennifer Sass

**Affiliations:** Natural Resources Defense Council Washington, DC E-mail: jsass@nrdc.org

Science plays a critical role in arbitrating the safety and efficacy of consumer and industrial products. Just as critical is the role of scientific journals, which can give published science the imprimatur of independence and therefore credibility. Richard Smith, an editor for the *British Medical Journal* for 25 years, has argued that the drug industry has become reliant on the publication of industry-funded studies that are published in major journals to give the research a “stamp of approval,” distribute the conclusions globally, and even attract media coverage (Smith 2005). “The quality of the journal will bless the quality of the drug,” Smith wrote. Effective disclosure policies play an important role in protecting journals from becoming unwitting agents of propaganda, distortion, corporate marketing, and other types of misinformation, thereby constituting an important cornerstone of their credibility and reputation.

In the summer of 2008, the Natural Resources Defense Council (NRDC) hosted a day-long workshop to discuss disclosure policies for health science journals. Participants included journal editors, other journal staff, academic scientists, scientific consultants, ethics experts, and publishing-house representatives. The product of the workshop is an NRDC report (Sass 2009) in which we recommend key elements of an effective and enforceable disclosure policy to guide journals in shaping and refining useful disclosure policies. Here I provide a summary of the results of that workshop.

Journal staff should clearly identify how far back they wish their journal policy on competing interests to extend, considering what is appropriate for the journal. Under no circumstances should policies be limited only to current conflicts. The public statement should be no more than a few sentences and should include relevant patents, employment, collaborations, consulting, and so on, that could be seen as a possible competing interests. If no competing interests are disclosed, then this should be stated in the public statement.

The disclosure policy should address both financial and nonmonetary relevant competing interests. If authors declare competing interests to the journal, they should also declare approximate monetary value.

A strong competing interest policy requires that all authors declare that the manuscript is free from the sponsor’s influence. Many journals already incorporate some kind of statement regarding the role of the funding source. If there was no such involvement, then a statement to that effect could be provided.

Disclosure policies should apply to all authors and should also be extended to peer reviewers and journal editors, where “editors” include all of the people who are involved in the peer-review and decision-making processes about articles; this would exclude others involved in the editing process, such as copy editors, managing editors, and proofreaders. For editors and for authors of review articles and editorials, a greater level of scrutiny and public disclosure to the reader is warranted because these circumstances may influence the selection of the science that will be reviewed or published.

As a rule of thumb, disclosures must include any financial interests that could constitute a potential source of bias—or of perceived bias—in the eyes of the general public, the media, the scientific community, peer reviewers, or editors. The NRDC (Sass 2009) recommends the following language for requesting disclosures:

All financial interests must be disclosed. This includes but is not limited to employment, clients, honoraria, travel expenses, grants, and litigation support. The approximate monetary value of any financial interests must be declared and should distinguish between funding for research and monies paid to the author. Disclosures should include anticipated future competing interests, and past competing interests going back a minimum of three years. Any other competing interests or potentially competing interests, financial or other that, when known to the public could compromise the standing or integrity of the journal, peer reviewers or author should be disclosed. The journal editors will then decide how best to manage these.

The public statement (as well as the detailed listing of competing interests) should be written in language such that the average person would be able to identify a potential competing interest. A mere listing of funding sources for a study or the author’s salary or honoraria is not adequate if most readers are not able to establish the link to a potential source of bias.

All participants of the NRDC workshop agreed that enforcing a policy is critical to making it effective. All workshop participants agreed that disclosure policies boil down to a mechanism in which disclosure enables peer reviewers and other professionals in the same field to alert journal staff if authors fail to disclose appropriately. If an author is found to have failed to disclose competing interests appropriately and further refuses to disclose in response to a request by the journal staff, punitive methods could include retraction of the article or banning the author from publishing in that journal for a specified length of time. *EHP* strengthened its policy in 2004 to allow a 3-year ban for authors found to “have willfully failed to disclose a competing interest” (Environmental Health Perspectives 2009). The journal editor could reject the paper at any stage for failure to disclose, and in cases where that failure appears to be deliberate or suspicious, the editor should consider referring the possible infraction to the senior author’s institutional superior for further investigation, along with any relevant documentation.

Because the critical issue underlying disclosure is the reliability of the information being reported in a manuscript, NRDC workshop participants also suggested that journals could conduct random audits in which authors of a randomly selected article are asked by the journal for a more detailed data report, potentially including submission of raw data.

Many of the most respected scientific and medical journals have instituted effective, practical disclosure policies. Although these policies may differ from journal to journal, they share certain elements that can be adapted to the specific needs of any journal. Given the importance of the scientific literature in guiding consumer and industrial health policy, it should be the goal of all journals to ensure integrity in the articles they publish. Strong disclosure policies are a critical tool for achieving this goal.

## Figures and Tables

**Figure:**
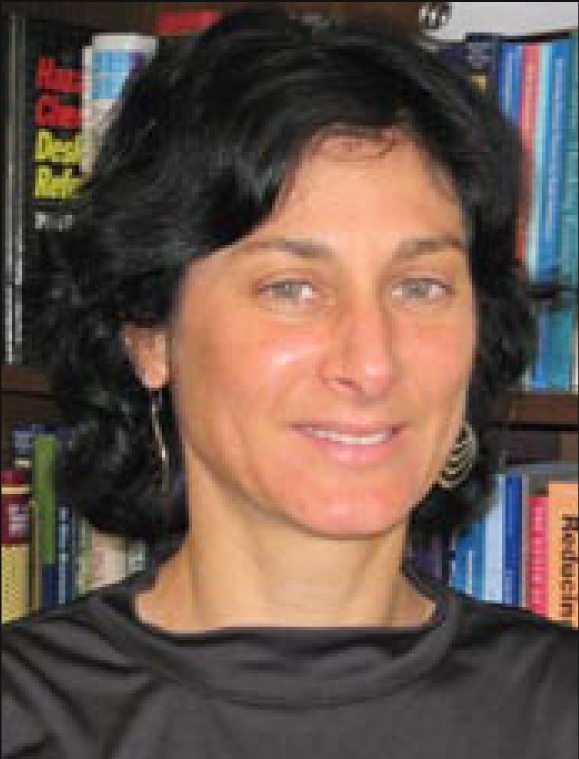
Jennifer Sass
